# Simple and cost-effective microfabrication of flexible and stretchable electronics for wearable multi-functional electrophysiological monitoring

**DOI:** 10.1038/s41598-021-94397-w

**Published:** 2021-07-20

**Authors:** Chae Hyun Kim, Dong Hyeon Lee, Jiman Youn, Hongje Lee, Joonsoo Jeong

**Affiliations:** 1 Medical Research Institute, Pusan National University, Yangsan, 50612 Republic of Korea; 2 School of Mechanical Engineering, Pusan National University, Busan, 46241 Republic of Korea; 3 Information Convergence Engineering, Pusan National University, Yangsan, 50612 Republic of Korea; 4 Department of Nuclear Medicine, Dongnam Institution of Radiological and Medical Sciences, Busan, 46033 Republic of Korea; 5 School of Biomedical Convergence Engineering, Pusan National University, Yangsan, 50612 Republic of Korea

**Keywords:** Health care, Engineering

## Abstract

The fabrication of flexible and stretchable electronics is a critical requirement for the successful application of wearable healthcare devices. Although such flexible electronics have been commonly fabricated by microelectromechanical system (MEMS) technologies, they require a specialised equipment for vacuum deposition, photolithography, and wet and dry etching. A photolithography-free simple patterning method using a desktop plotter cutter has been proposed; however, the metal formation and electrode opening still rely on the MEMS technology. To address this issue, we demonstrate a simple, rapid, cost-effective, and a complete microfabrication process for flexible and stretchable sensor platforms encompassing conductor formation and patterning to encapsulate and open sensing windows, which only require an economic plotter cutter and readily available supplies. Despite its simplicity, the proposed process could stably create microscale features of 200 μm wide conductor lines and 1 mm window openings, which are in the useful range for various wearable applications. The feasibility of the simple fabrication of multi-functional sensors for various physiological monitoring applications was successfully demonstrated in electrochemical (glucose), electrical (electrocardiogram), mechanical (strain), and thermal (body temperature) modalities.

## Introduction

The recent advancements in flexible and stretchable electronics have paved the way for biomedical diagnostic and therapeutic applications. Unlike the conventional rigid electronics, flexible and stretchable devices can conform to the soft and curved human skin and deform with the dynamic movement of the human body without detachment or fracture, thus enabling intimate and stable interfaces to the physiological signal sources. One of the most promising applications of such devices is in wearable electronics, in which biosensors integrated on thin flexible polymeric substrates are mounted on the skin for continuous monitoring of various physiological information^1–7^. Wearable electronics are synergistically incorporated with wireless communication capabilities, such as Bluetooth, ZigBee, WiFi, and near-field communication, to enable untethered and continuous health monitoring without interrupting the daily lives of users.

Epidermal electronics, one of the successful examples, involves mechanical skin-like substrates integrated with multiple physiological sensors and wireless functionalities^8^. Such epidermal electronics and typical state-of-the-art wearable sensors are commonly fabricated based on standard microelectromechanical system (MEMS) technologies, including vacuum deposition, photolithography, and wet/dry etching. Although they are effective for a high patterning resolution and potential batch production, several challenges hinder further extension of the benefits of new technologies. Conventional microfabrication processes require special equipment and cleanroom facilities, including a mask aligner, plasma etcher, physical and chemical deposition systems, and multiple photomasks, which may not be affordable/accessible for some institutions and regions. The MEMS process is also time-consuming in that it requires vacuuming, film growth, and etching for a sequential layer-by-layer construction. The necessity of carrier wafers for compatibility with the MEMS equipment, regardless of the substrate materials, not only limits the size of the devices and batches, but also hinders the potential use of new mass-production technologies such as roll-to-roll processes.

As the typical feature sizes required for wearable devices [tens to hundreds of micrometres (μm)] are generally larger than those of MEMS structures (a few to tens of μm), the cost and time inefficiencies of conventional microfabrication processes could be alleviated by introducing low-cost and rapid patterning technologies that only require an affordable desktop equipment, and yet provide sufficient precision for target applications. As an alternative, a photolithography-free process using plotter cutters for patterning metal sheets or plastic substrates has been demonstrated^9^. The cutting method has mostly been employed for a rapid prototyping of microfluidic channels by either creating negative channel moulds for casting or directly engraving channel structures on polymer sheets^10–15^. Wearable or in-vitro biosensors have also utilised the ‘cutting’ process as a simplistic metal patterning scheme by creating stencil masks for a silkscreen of conductive ink or shadow masks for selective metal deposition^16–24^. Kirigami patterning is another recent demonstration of such a technology for the rapid production of stretchable wearable electronics^25^. A comprehensive application of the cutting process for wearable flexible electronics has been proposed as a ‘cut-and-paste’ method^26^, in which a thin gold layer evaporated on a temporary substrate is outlined by a desktop cutting machine before being transferred to target substrates such as medical dressing or tattoo papers. This method has been successfully extended to other applications, including monitoring of electrocardiogram (ECG), electromyogram (EMG), electrooculogram, seismocardiography, bioimpedance, skin hydration, skin deformation, and skin temperature^4,26–31^. Copper foils or copper-clad substrates have also been employed for similar processes, particularly for wireless functions^32,33^.

Despite the successful demonstration of a simpler and faster prototyping compared to conventional microfabrication processes, the abovementioned cutting methods still have several limitations, thus hindering their wider use in wearable biomedical applications; these limitations include (1) the lack of selective encapsulation methods for creating sensing windows, (2) use of vacuum deposition for the gold layer, and (3) limited types of target substrates to adhesive materials such as medical tapes or tattoo papers. First, encapsulation with selective opening of sensing electrodes is essential for electrical or electrochemical measurements. This issue becomes more critical in monolithically integrated multi-functional biosensors for electrical isolation and minimised interference among sensors. This is typically achieved by spraying liquid encapsulants with manual masking^30,31^. In this study, we demonstrate a complete ‘cut-and-paste’ plus ‘cut-and-insulate’ method by utilising the same low-cost cutting plotters. Second, although the plotter cutter has replaced photolithography, the metallisation step still relies on MEMS vacuum deposition^26,28,30,34^, which may partially weaken the motivation for the introduction of the low-cost patterning technique. Copper foil or copper-clad substrates can be used^32,33^, but may not be suitable for direct contact with the skin owing to toxicity. We propose thin gold leaves as a cost-effective and rapidly prepared conducting layer with a good compatibility with the cut-and-paste method. A skin-transferred sensor has been fabricated from gold leaves^35^; however, it may not be mechanically robust without a polymeric substrate^27^. Gold leaves have been successfully utilised in the fabrication of implanted neural interfaces^36^. Finally, we demonstrate the transfer printing of cut patterns onto nonsticky substrates using a chemical treatment and half-curing of polydimethylsiloxane (PDMS). Considering the applicability of PDMS in various biomedical applications, this would facilitate the integration with other modalities such as microfluidics or optics, thus extending the usefulness of the proposed low-cost process.

Therefore, in this study, we propose a simple and rapid, and yet a complete fabrication process for wearable flexible and stretchable electronics, including conductor formation, conductor patterning, encapsulation, and window opening, which are based on a low-cost manufacturing using a plotter cutter. The feasibility and effectiveness of the proposed process are demonstrated through a multi-functional epidermal sensor patch integrated by mechanical (strain), electrochemical (sweat glucose), electrical (ECG), and thermal (skin temperature) modalities.

## Results

### Simple and low-cost microfabrication process

**Figure 1:**
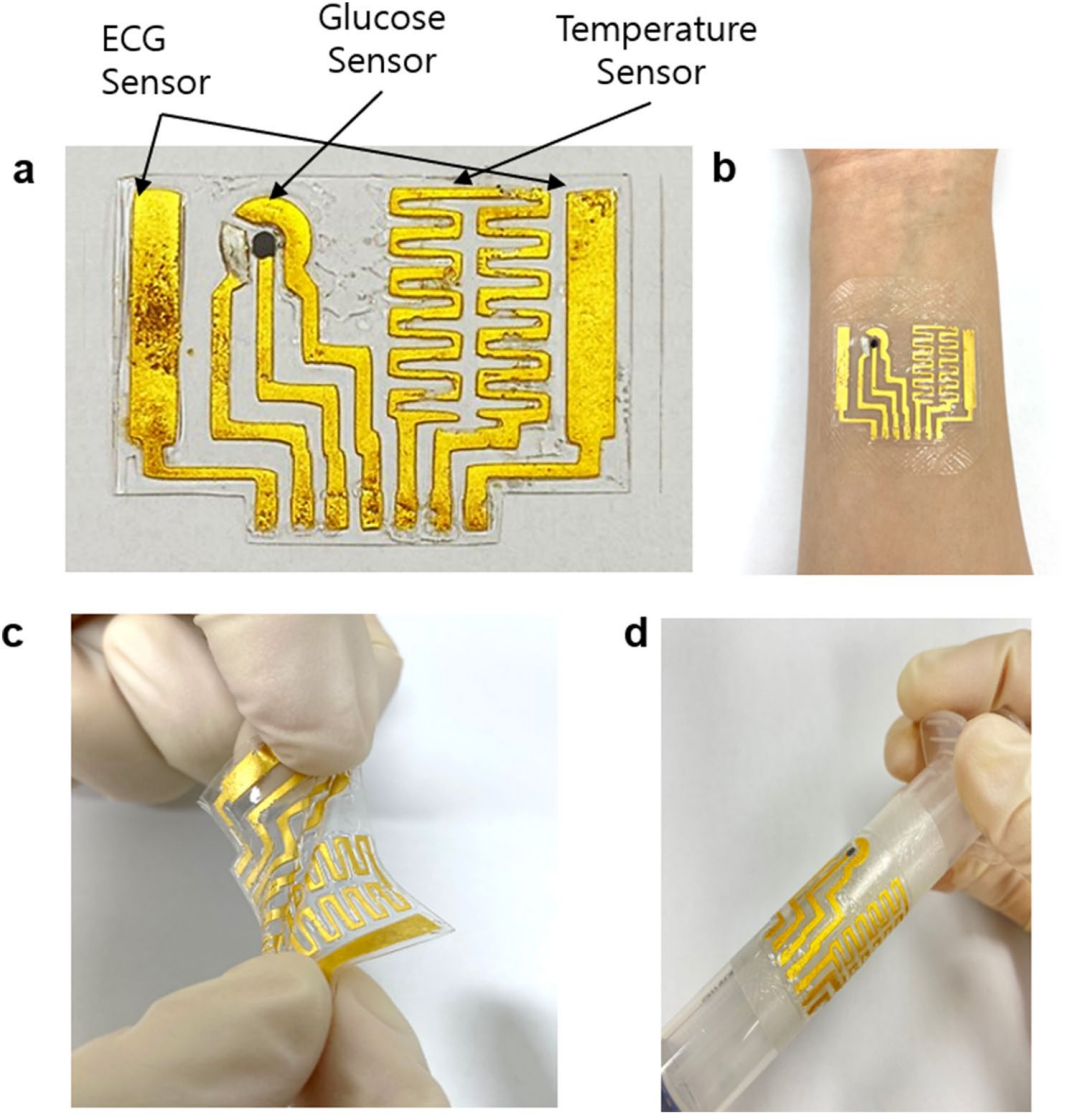
Fabricated flexible and stretchable wearable patch-type sensor. (**a**) Multi-functional physiological monitoring capability for ECG, glucose, and temperature sensing on a 40 mm × 25 mm PDMS patch. (**b**) The sensor is conformally attached on the skin around the wrist. (**c**) The sensor can be twisted and stretched with mechanical robustness. (**d**) The sensor can be conformally wrapped around a syringe with a diameter of 17 mm.

Figure 1 presents the fabricated flexible and stretchable wearable patch-type sensor (40 mm × 25 mm) with a multi-functional physiological monitoring capability. As a proof-of-concept, the device is designed to integrate a sweat-based glucose sensor, ECG sensor, and temperature sensor onto a 250 μm thick PDMS substrate (Fig. 1a). The overall patch size was determined by the ECG electrode size (23 × 5 mm^2^) and spacing (35 mm) optimized for both compactness and signal quality based on literature^28,37,38^. The entire device can be conformally attached to the curved skin around the wrist (Fig. 1b). The PDMS substrate and gold-leaf metal traces provide flexibility and stretchability with mechanical robustness such that they can be stretched, twisted (Fig. 1c), and conformably wrapped around a syringe (*r* = 17 mm) (Fig. 1c). The target substrate is not limited to PDMs, but can be readily transferred onto other soft materials widely used in biomedical applications such as polyethylene terephthalate (PET) and medical dressing as demonstrated in Supplementary Fig. S1.

**Figure 2:**
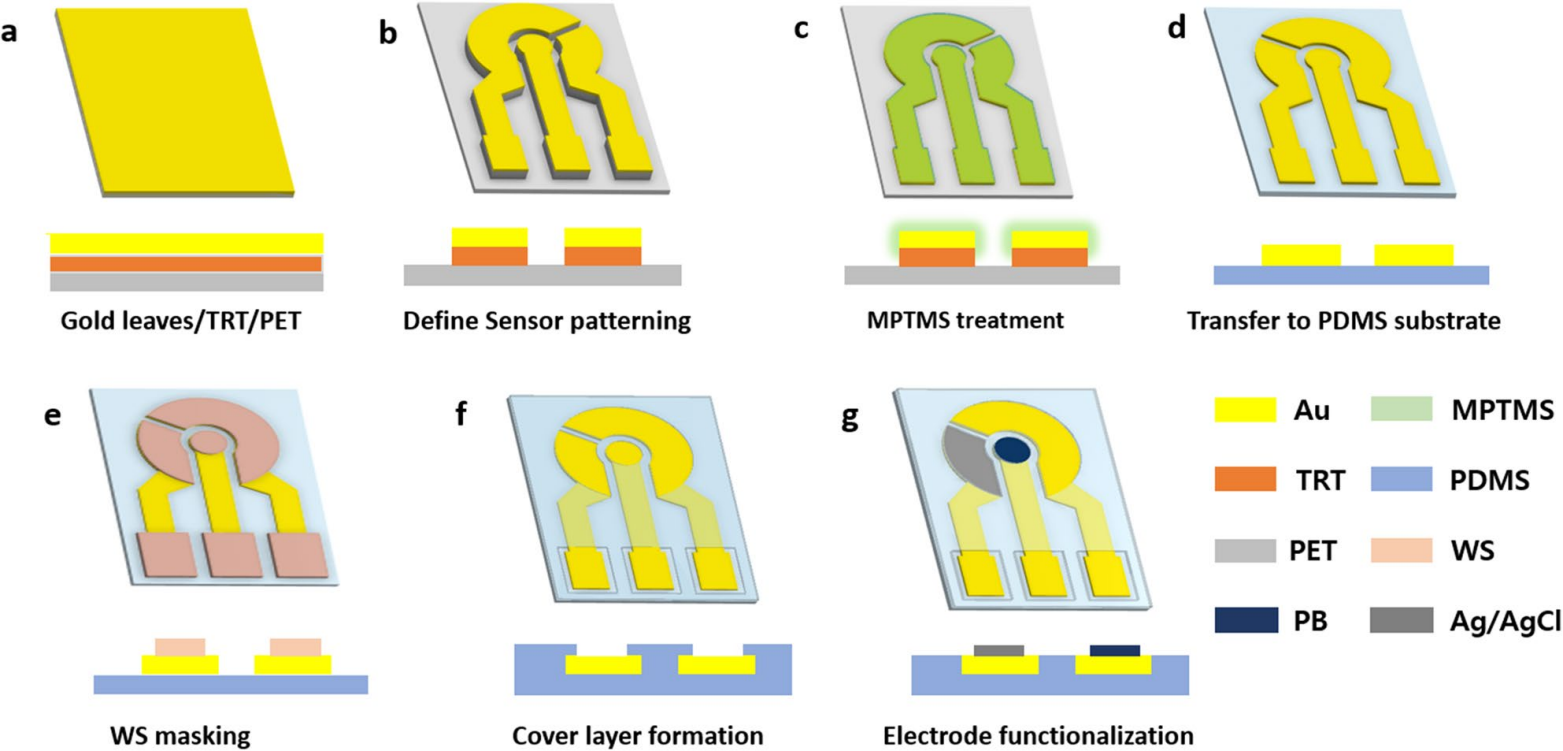
Simple and cost-effective microfabrication process for a flexible and stretchable sensing platform without MEMS equipment. (**a**) Gold leaves mounted on the PET carrier film via the TRT, (**b**) sensor pattern defined by the plotter cutter, (**c**) gold surface treated by MPTMS, (**d**) transfer to the half-cured PDMS substrate, (**e**) sacrificial WS mask pattern transferred on the gold pattern, (**f**) spin coating of the cover layer and dissolution of the WS mask, and (**g**) optional electrode functionalisation with stencil masks.

A simple and low-cost microfabrication process using only an affordable desktop equipment without requiring any cleanroom facilities such as vacuum deposition, photolithography, and plasma etcher is illustrated in Fig. 2. A commercially available 24-K gold film (referred to as ‘gold leaf’, which is ~ 100 nm thick) is mounted on a PET film using a thermal release tape (Fig. 2a) and cut into the desired sensor pattern using a plotter cutter machine (Fig. 2b). After peeling off the cut-out area, the sample was treated in a (3-mercaptopropyl)trimethoxysilane (MPTMS) solution^39,40^ (Fig. 2c) before being transferred onto a half-cured PDMS substrate (Fig. 2d). Both MPTMS treatment and half-curing of the bottom layer played a critical role in achieving a strong adhesion between the gold leaf layer and PDMS substrate, as discussed in the following section. A water-soluble (WS) tape mounted on a separate PET film carved by the same plotter cutter was transferred onto the metal pattern to serve as a masking layer during the spinning of the cover PDMS layer (Fig. 2e). The soft wearable sensor patch with selective encapsulation for individual electrical access was completed after dissolving the WS structure (Fig. 2f). Optionally, selective electrochemical functionalisation can be performed using PET stencil masks, also prepared by the plotter cutter, for the squeezing of inks or drop casting of the enzyme (Fig. 2g). The completed step-by-step description and more experimental details are presented in “Methods” section and Supplementary Fig. S2.

### Characterisation of the fabrication process

**Figure 3:**
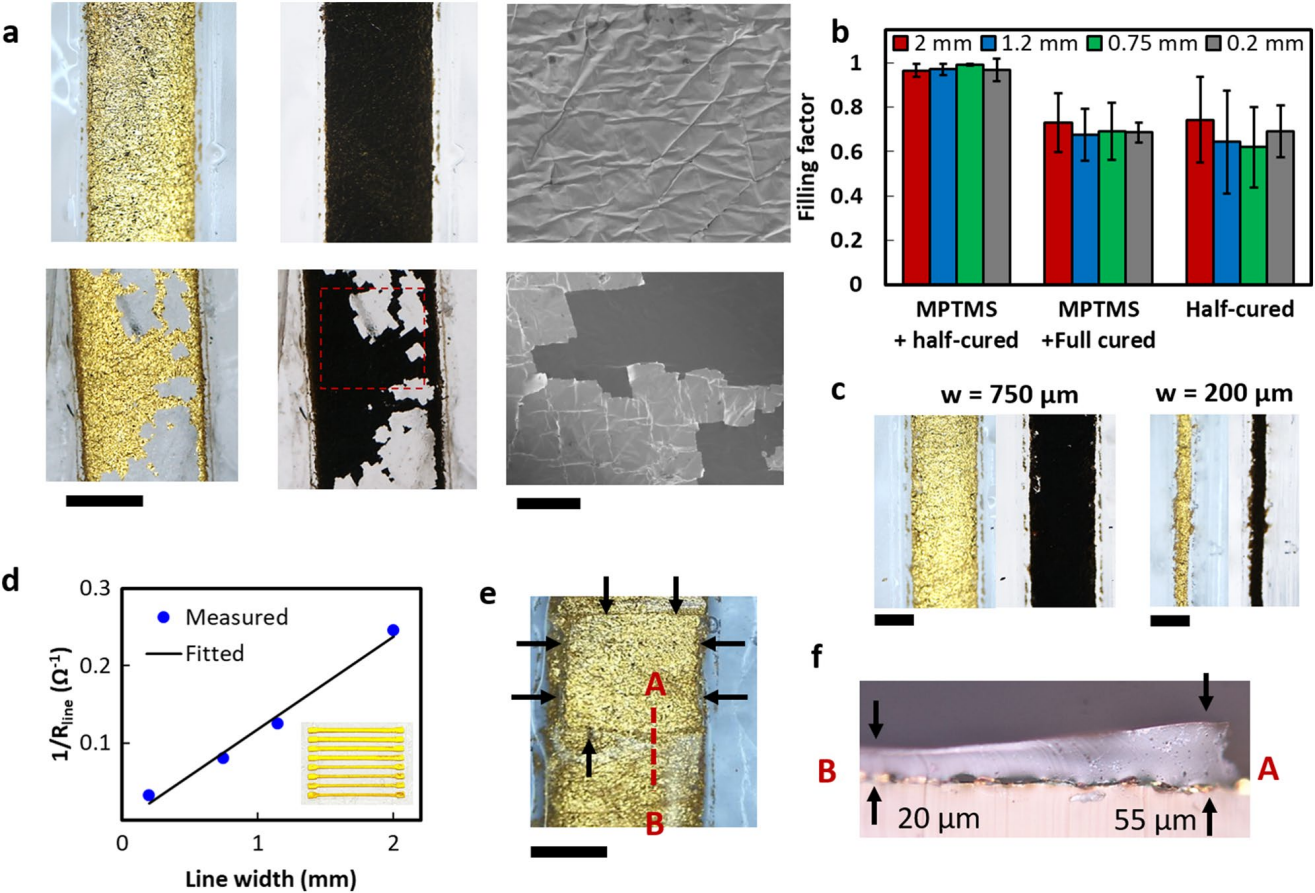
Characterisation of the proposed simple microfabrication process. (**a**) Top-lighted (left) and back-lighted (centre) and SEM (right) images of the gold lines produced by the ‘MPTMS and half-cured PDMS’ scheme (upper row) and ‘MPTMS and fully cured PDMS’ or ‘no MPTMS and half-cured PDMS’ (bottom row) (scale bar: 1 mm for microscopy images and 50 µm for SEM images). (**b**) Fill factor chart comparing the integrity of the fabricated gold patterns with different surface treatments and line widths. (**c**) Stably producible line width by the proposed process; top-lighted and back-lighted images of 0.75 mm (left) and 0.2 mm (right) lines (scale bar: 0.5 mm). (**d**) Linear relationship of the resistance with the line width. (**e**) 1 mm sensing windows opened above the gold pattern (scale bar: 0.5 mm) and (**f**) its cross section at the edge (upward curve).

The patterning of the conductor and formation of opening windows using the proposed fabrication process were characterised, as shown in Fig. 3. The surface treatment of the gold and PDMS significantly affected the adhesion between them and the consequent integrity of the transferred metal tracks (Fig. 3a,b). The gold patterns treated by MPTMS and transferred to the half-cured PDMS substrate resulted in the best line quality with void-free and crack-free gold surfaces, as observed by top-lighted and back-lighted microscopic images and scanning electron microscopy (SEM) images (Fig. 3a, upper row). Gold patterns that were either treated by MPTMS and transferred onto a fully cured PDMS substrate or those that were not treated by MPTMS and transferred onto a half-cured PDMS substrate produced similar results with a poorer adhesion than that of the ‘MPTMS + half-cured’ case, with a significant content of voids formed by delamination of gold patterns due to the lack of adhesion (Fig. 3a, bottom row). The line integrity was quantified by the ‘fill factor’ from subimages sampled from back-lighted images (i.e., dashed red box in Fig. 3a), which is defined as the ratio of the dark area (densely packed gold) to the entire area of the samples with different surface treatments (Fig. 3b and Supplementary Fig. S3). The ‘MPTMS + half-cured’ scheme consistently created void-free lines with a fill factor of approximately 100% for all line widths (0.2, 0.75, 1.2, and 2 mm), while the other two treatments led to fill factors of ~ 60% with a larger variance.

Using this ‘MPTMS + half-cured’ process, gold lines with a minimum width of 0.2 mm could be stably fabricated, as shown in Fig. 3c. The plot of *R*^−1^ versus the line width showed a linear relationship (*R*^2^ > 0.98), indicating uniformity of the gold patterns (Fig. 3d). The WS tape pattern cut by the plotter cutter successfully served as a masking structure during the spinning of the cover layer and created opening windows after the dissolution. The smallest opening size that could be stably and reproducibly created was 1 mm, as shown in Fig. 3e (the perimeter of the opening is indicated by arrows). Cross-sectional microscopy images revealed that the surface tension between the liquid PDMS resin and sidewall of the WS masks created an upward meniscus curve (at most ~ 35 μm above the ~ 20 μm thick layer) around the edge of the opening windows (Fig. 3f). Although this curve may increase the distance between the sensor and target tissue, it is considered acceptable in most applications considering the aspect ratio of the openings (diameter > 1 mm, height of tens of μm).

Conducting lines with widths of 0.2 mm and 1 mm opening windows are larger than general MEMS structures, but this range of micropatterns is still acceptable for various wearable electronics, in which the lead count and area restriction are not as harsh as in MEMS applications.

### Electromechanical characterisation as a strain sensor

**Figure 4:**
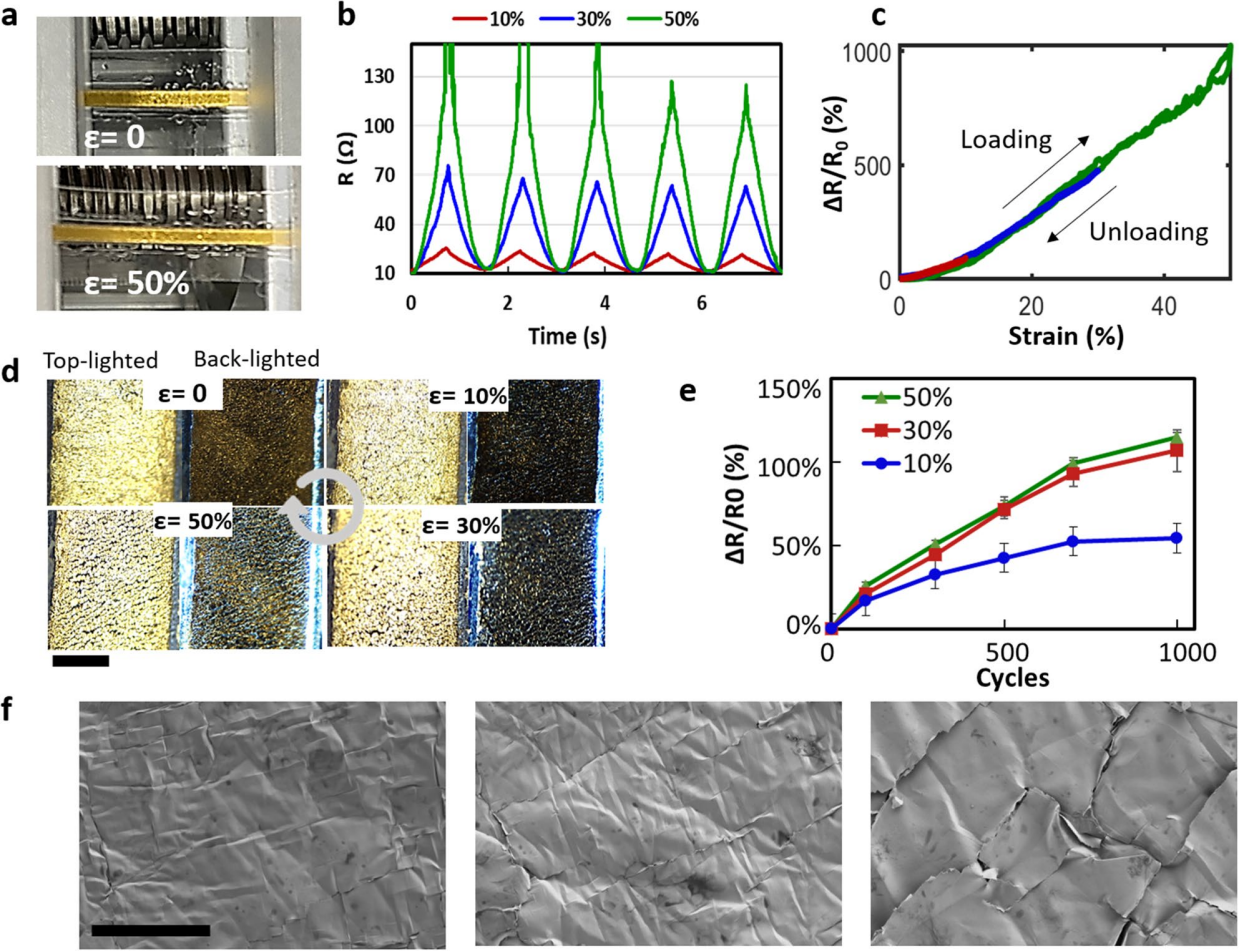
Mechanical characterisation of the strain sensor. (**a**) Strain sensor elongated up to a strain of 50% using a custom-built strain testing setup. (**b**) Resistance changes during the first five stretching/relaxation cycles of the strain sensor with different strains, showing a stabilising behaviour. (**c**) Δ*R*/*R* curves versus the strain with maximum elongations of 10, 30, and 50%, showing the linear response to the strain. (**d**) Top-lighted and back-lighted microscopic observation of the gold surfaces in the course of stretching from 0 to 50% (scale bar: 1 mm). (**e**) Baseline resistances at relaxed states after completing an increasing number of cycles for different maximum strains. (**f**) SEM images of gold surfaces before (left) and after 1000 repetitions of application of strains of 10% (centre) and 50% (right) (scale bar: 50 μm).

The mechanical characterisation of the fabricated metal tracks upon tensile elongation and their application as a strain sensor are shown in Fig. 4. For the strain characterisation, a strain sensor with lengths of 26 mm and width of 2 mm created by the same fabrication process were subjected to repeated stretching (strain *ε* = 10, 30, and 50%) and relaxation, while their line resistances were monitored (Fig. 4a). The test was performed using a custom-built setup consisting of a linear rail, step motor, Arduino Uno board for motor control, and digital multimeter (Keithley 6510) The first five stretching–releasing cycles under strains of 10, 30, and 50% are shown in Fig. 4b. The sensors under the three different maximum strains exhibited good linearity, recoverability, and reproducibility. Notably, a ‘stabilising’ behaviour was observed, wherein the strain-dependent resistance change was stabilised after approximately three cycles for all strain values. Representative relative resistance changes (Δ*R*/*R*_0_) after stabilisation with respect to elongation cycles of varying maximum strain from 10 to 50% are shown in Fig. 4c. The Δ*R*/*R*_0_ curve showed an almost linear response (*R*^2^ > 0.96) to the strain with a negligible hysteresis. The slope of the curve, referred to as gauge factor [*K* = (Δ*R*/*R*_0_)/*ε*] for the determination of the sensitivity of the strain sensor, was calculated to be ~ 20. The high linearity of the sensor is beneficial for diverse applications with sensitive and precise strain measurements. Despite the small resistance fluctuation at strains of approximately 50%, the proposed sensors could monitor tensile strains up to 50%, which is suitable for sensing human motions compared to commercially available metallic gauges. Microscopic images were acquired during the course of stretching and relaxation (Fig. 4d) to facilitate the understanding of the electromechanical behaviour of the fabricated strain sensor. As tensile strain is applied to the sensor, the densely packed gold films experience the formation of cracks dominantly in the direction perpendicular to the stretching. More cracks were developed under a higher elongation, which implies that the increasing resistance with the strain is attributed to the loss of longitudinally conducting paths owing to the cracks in the perpendicular direction. However, when the sample was relaxed to zero strain, the cracks were closed, and the initial current pathways were reconnected, which is consistent with the resistance at the relaxed position returning to the original value.

To assess the number of repetitions during which the crack formation/closure occurs reversibly, the test samples were subjected to repeated stretching/relaxation up to 1000 cycles with varying strain (10, 30, and 50%). Figure 4e shows the baseline resistance at the relaxed position after the stretching/relaxation cycles with varying maximum strain. When the strain of 10% was repeatedly applied, the baseline resistance gradually increased with the repetition number, and then levelled off, reaching 54% above the initial resistance after 1000 cycles. Similar trends were observed for the repeated application of the strain of 30% and 50%, with increased baseline resistances up to 107% and 114%, respectively, after 1000 cycles. The baseline resistances increased but stabilised after ~ 1000 cycles, which suggests a predictable behaviour for strain sensor applications. Greater elongations induced higher baseline resistances, presumably due to the fact that more permanent cracks are produced by greater strains, which was confirmed by SEM observations. Figure 4f compares the gold surface before (left) and after the 1000 cycles of strains of 10% (centre) and 50% (right). As expected, more and longer cracks were observed at the gold surface that underwent a higher strain. The repeated stretching/relaxation cycles induced permanent cracks, which could not be ‘healed’ even after the relaxation. Although the cracks are ‘closed’ in the relaxed state connecting the separated areas without leaving any voids, they are considered responsible for the partial breakdown of conducting paths, which increased the baseline resistance.

These findings of the electromechanical evaluation suggest that the proposed fabrication process can produce an efficient strain sensor with predictable and linear responses for strains < 50%.

### Characterisation as an electrochemical sensor

**Figure 5:**
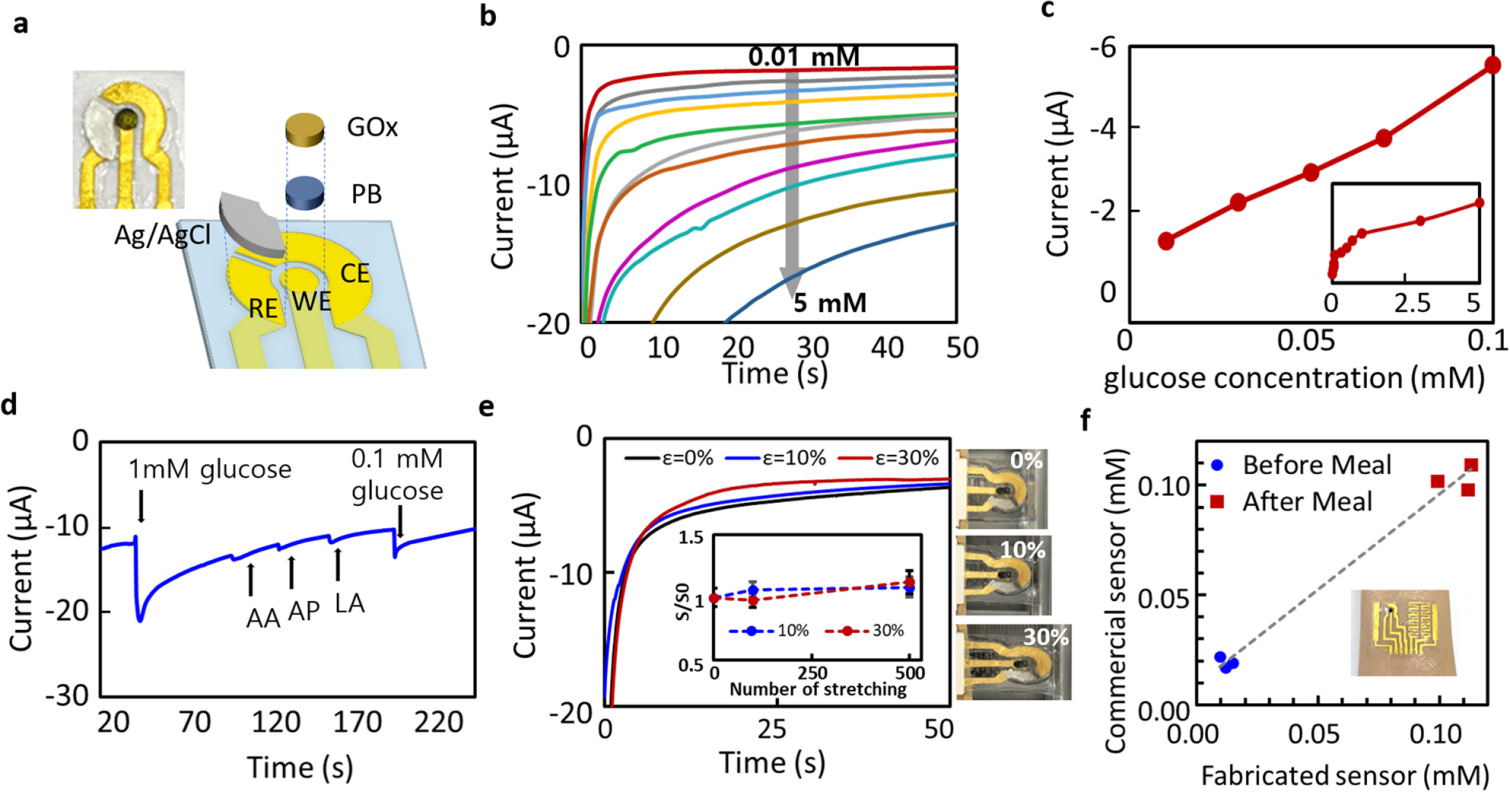
Characterisation as an electrochemical sensor for glucose monitoring. (**a**) Schematic of the electrode functionalisation of three electrodes using GOx and PB on the WE and Ag/AgCl on the RE. (**b**) CA curves of the glucose sensor in response to varying glucose concentration from 0.01 to 5 mM. (**c**) Calibrated curve of the response current versus the glucose concentration showing a linear relationship within the range of human sweat (inset shows the current response to the glucose concentration up to 5 mM). (**d**) CA curve of the sensor in the presence of physiological molecules such as AA, AP, and LA, showing a good selectivity towards glucose molecules. (**e**) CA responses to 0.1 mM glucose concentration from the patch sensors in rest states and stretched by 10 and 30%. Inset shows the relative sensor responses (S/S_0_) after repetitive stretching cycles of 10% and 30%. (f) Correlation of glucose concentration from human sweat measured and calibrated by the fabricated sensor (x-axis) with those by commercial glucose sensing strips (y-axis) before and after meal.

The feasibility of multi-functional wearable sensor applications including sweat-based glucose, ECG, and temperature sensors for physiological monitoring for electrochemical, electrical, and thermal modalities, respectively, was demonstrated. Glucose monitoring from sweat has attracted increasing attention for noninvasive and continuous glucose monitoring as an alternative to conventional glucose sensing from the blood^41–45^. Figure 5a illustrates the electrode functionalisation through immobilisation of the enzymatic glucose oxidase (GOx) layer on top of the working electrode (WE) and deposition of Ag/AgCl on top of the reference electrode (RE) (see “Methods” section for the detailed functionalisation process). GOx oxidises the glucose in sweat to produce hydrogen peroxide (H_2_O_2_), which is reduced by a redox mediator layer, typically Prussian blue (PB), so that the concentration can be quantified by measuring the current by constant-potential chronoamperometry (CA). Figure 5b shows CA response curves at a potential of − 0.4 V for increasing glucose concentration from 0.01 to 5 mM. Well-defined current signals corresponding to increasing glucose concentrations were observed with stable responses obtainable within 30 s. The calibration plot in Fig. 5c indicates a linear relationship between the output current and glucose concentration in the range of 0.01 to 0.1 mM, the typical glucose levels in human sweat^42^. The inset shows the calibration curve in the whole range, with an almost linear trend up to 0.1 mM, which levels off above a glucose concentration of 1 mM. The selectivity of the sensor was investigated in the presence of physiological molecules in sweat, including ascorbic acid (AA), uric acid (UA), and acetaminophen (AP). Compared to the response of glucose droplets (1 and 0.1 mM), the CA curve in Fig. 5d presents negligible interference signals when 1 mM of UA, AP, and AA were sequentially dropped. Considering that the concentrations of these molecules in human sweat (AA, AP: 1 μM; UA: 5 μM) are considerably lower than 1 mM used in the test, this observation implies an excellent selectivity of the sensor towards glucose molecules.

The sensor could stably produce CA responses to 0.1 mM glucose concentration under uniaxial stretching of 10% and 30% when compared to the response at the rest state (ε = 0%) as shown in Fig. 5e. Elongation of the sensor up to 50% resulted in delamination of the relatively rigid functionalizing layers (drop-casted enzyme and silk-screened PB and Ag/AgCl layers) from the underlying stretchable gold layer, leading to unstable CA response. The inset in Fig. 5e shows sensing performance after repetitive stretching cycles, indicated by the relative sensitivity (S/S_0_), where S and S_0_ are the CA responses to 0.1 mM glucose before and after the repeated strain, respectively. The sensor demonstrated relatively stable responses without significant degradation of initial sensitivity after repeated stretching up to 500 cycles, despite slightly increased deviation which is attributed to randomly generated microcracks on the gold film as shown in Fig. 4. The glucose sensor has been tested on human body and compared to the measurements from commercial glucose sensing strips (Accu-Chek, Roche). Figure 5f demonstrates the glucose concentration in human sweat accumulated during exercise before and after meals measured using our patch sensor (x-axis) and commercial sensors (y-axis). High linear correlation (R > 0.98) of the calibrated glucose concentrations from both sensors confirm the feasibility of the proposed patch sensor for glucose monitoring from human sweat.

### Characterisation as an electrical sensor

**Figure 6:**
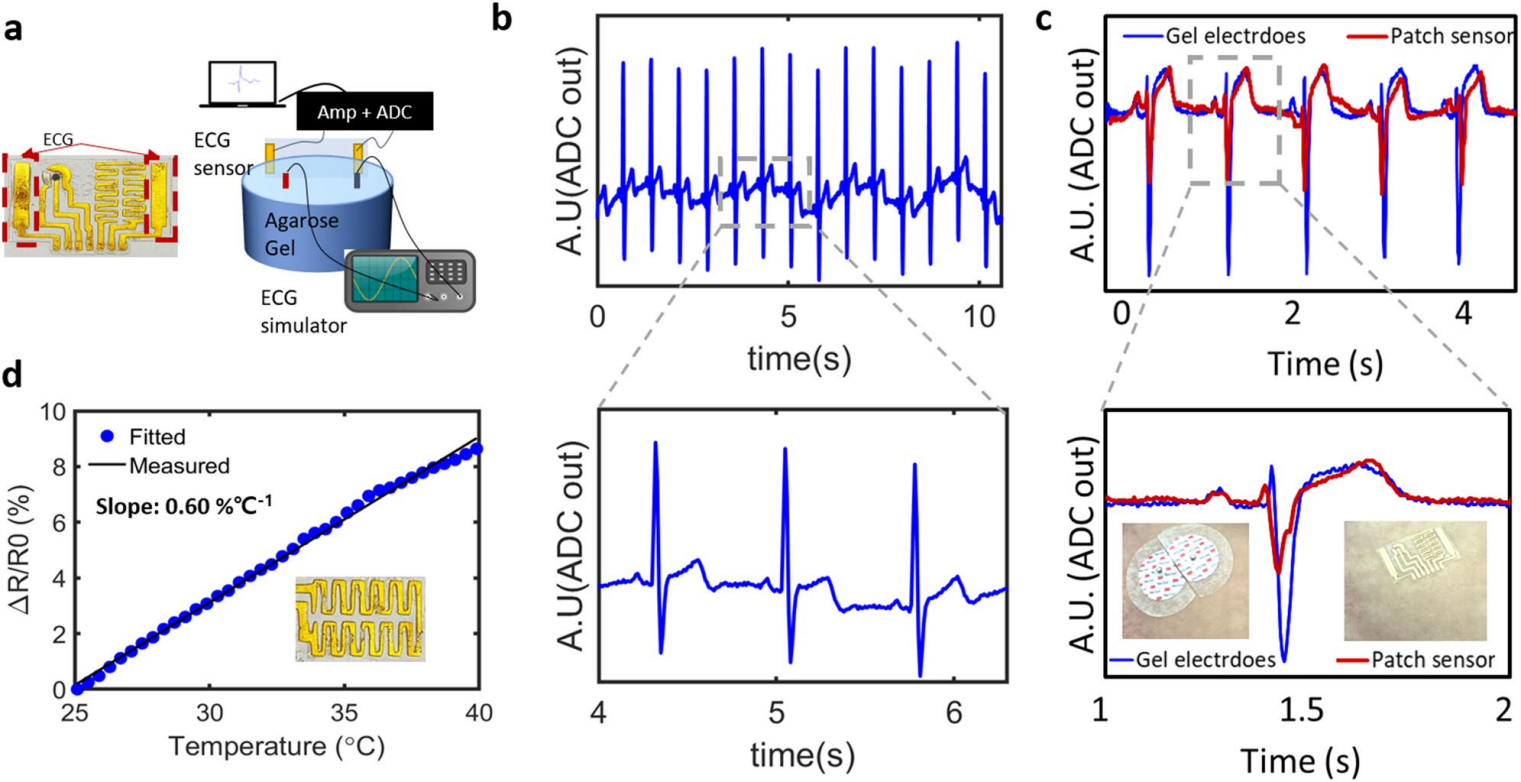
Electrical and thermal characterisations for ECG and temperature monitoring. (**a**) Schematic of the measurement of ECG signals using a chest phantom and ECG simulator. (**b**) Recorded ECG signals using the proposed ECG sensor and magnified view clearly showing PQRST waveforms. (**c**) ECG signal measured from the patch sensor attached on the chest of human subject, compared to the signal from conventional gel-type electrodes. (**d**) Linearly increasing resistance of the temperature sensor.

The electrical physiological monitoring capability was evaluated using an ECG and temperature sensor. As shown in Fig. 6a, the ECG sensor was tested in a chest phantom consisting of a cylindrical block of agarose gel and pseudo ECG signal generated by an ECG simulator (MS400, Contec). The ECG measured from the surface of the phantom ~ 20 mm away from the signal source faithfully recorded the ECG signals, as shown in Fig. 6b. A magnified view of the recorded ECG signals clearly displays the captured PQRST waves. The ECG signals were also recorded from a human subject by attaching the fabricated patch sensor on the chest near heart as shown in Fig. 6c. Conventional gel-type electrodes (Red Dot, 3 M) were placed on the identical location as a control, after trimmed to ensure the same inter-electrode distance as the patch sensor (3.5 mm). The patch sensor could stably capture the ECG waveforms with clearly discernible PRST peaks, showing similar signal quality to the conventional electrodes. While the amplitude of S-peak is smaller, which is attributed to the higher interface impedance of the patch sensor than gel electrodes as a result of smaller surface area, these results confirm the feasibility of the proposed patch sensor as a wearable ECG sensor platform.

The resistance measurements in response to the temperature showed an almost linearly increasing resistance (*R*^2^ > 0.99) with a slope of 0.60%/°C, as illustrated in Fig. 6d, which indicates the applicability of the proposed fabrication process in various applications of electrical sensing of physiological conditions.

## Discussion

Stretchable and flexible electronics have a great potential for successful clinical applications of various wearable healthcare devices. The microfabrication technology is crucial, particularly for such wearable devices, because a high degree of flexibility and stretchability is required to conform to the soft, curved, and dynamically active skin surfaces. Although MEMS-based microfabrication technologies have been commonly considered effective, they rely on expensive equipment and cleanroom facilities. We addressed this issue by a simple and cost-effective microfabrication process for flexible and stretchable electronics fabricated using only affordable desktop tools. The proposed process does not require any special cleanroom resources, such as vacuum deposition, photolithography, photomasks, plasma etching (nor plasma treatment), and wet etching. A hobbyist-level desktop plotter cutter (< $300) is the only required equipment and the economic gold leaves (< $0.1 per 1 cm^2^ sheet) are the most expensive supplies in this study. A spin coater (> $1000) may be useful for spinning the liquid prepolymer resin, but it is not essential if commercially available films of various materials with various thicknesses can be used. Despite its simplicity and low cost, the proposed process could stably create gold patterns with a width of 200 μm and was successfully demonstrated as a multi-parametric sensing platform for various physiological monitoring capabilities. The process could create an integrated sensor patch embedded by mechanical (strain), electrochemical (glucose), electrical (ECG), and thermal (temperature) sensors, the feasibility of which was successfully demonstrated. Although the conducting lines with widths of 0.2 mm are larger than general MEMS structures, this range of micropatterns is useful for various wearable electronics, in which the spatial restriction is not as severe as in general MEMS fields. Furthermore, unlike the restricted batch size in MEMS, which is limited by the carrier wafer size for compatibility of the equipment, the process proposed in this study is further scalable to large-area manufacturing such as roll-to-roll process, which suggests the potential advantage of a considerably lower production cost. The bottleneck against a larger-size production is the maximum film width of the plotter cutter, which is 30 cm in this study; however, larger plotter cutters are available in the market. Therefore, this simple and cost-effective microfabrication process is expected to be beneficial to developing regions of the world that have limited accessibility and/or affordability for cleanroom facilities.

The proposed fabrication process can offer extra advantages over the existing "cut-and-paste" methods. First, this method can provide a cutter-based simple and cost-effective fabrication process not only for metal patterning, but also for metal formation and selective encapsulation. This is an extra advantage over previous methods which typically have relied on MEMS metallization for vacuum deposition, and dry etching for selective encapsulation. Also, this method is applicable to a wider range of target substrates including PDMS layers via relevant surface treatment, other than the adhesive films used in the existing methods such as medical dressing and tattoo papers.

The feasibility of the proposed method was demonstrated for strain, ECG, glucose, and temperature sensing applications. The applications can be further extended to monitor other physiological signals including EMG, EEG, respiratory rate, skin hydration, oxygen saturation (SpO_2_), lactate, cortisol, and uric acid. As the fundamental sensing capability in electrical, electrochemical, mechanical, and thermal modalities was validated in this study, transition to other functionalities could be realised by a decent adaptation of the electrode design and electrode functionalisation.

One drawback of the sensor fabricated in this study is the increasing baseline resistance with the repeated strain cycles as a result of the irrecoverable crack formation. The increasing trend levelled off and saturated after 1000 cycles, such that the resistance changed in a predictable manner. Nevertheless, this implies the necessity for a major study to achieve a higher mechanical robustness of metal patterns. One possible solution would be the transfer of gold patterns onto a pre-strained PDMS substrate so that the out-of-plane buckling of gold layers in a relaxed position can provide a higher mechanical stability upon stretching^46,47^. Further reduction in the feature size below 200 μm is also plausible because an additional optimisation of the cutting process can be carried out by tuning several parameters of the cutting plotter, including the blade type, applied force, cutting speed, and repetition number. The transfer step can also be enhanced for finer pattern sizes by optimising the surface treatment of the gold sides and/or substrate side.

## Methods

### Fabrication

The simple and rapid fabrication process without photolithography and cleanroom facility is illustrated in Supplementary Fig. S2. A thermal release tape (TRT, Graphene Square, Korea) and gold thin film (Au, Hanil Gold Leaf Co., Korea) were sequentially laminated on top of a 100 μm thick PET film (Supplementary Fig. S2a). The sensor pattern was formed on the PET + TRT + Au film using a desktop plotter cutter (Cameo 3, Silhouette), followed by peeling off the cut-out area of TRT + Au from the PET substrate (Supplementary Fig. S2b). For a stable adhesion of the gold pattern onto the PDMS substrate, the gold pattern on the PET substrate was immersed in an MPTMS (175617, Sigma-Aldrich) solution (1 μL of MPTMS per 1 mL of ethanol) for 60 min (Supplementary Fig. S2c). After drying with nitrogen gas and leaving it at room temperature for 24 h (Supplementary Fig. S2d), the god pattern was transferred onto a separately prepared 200 μm thick PDMS substrate (Supplementary Fig. S2e). This PDMS substrate layer was prepared by spinning (1000 rpm) a PDMS mixture of prepolymer and curing agent (10:1 wt/wt, Sylgard 184, Dow Corning) on a glass wafer, followed by baking in an oven at 70 °C for 7 min to prepare a half-cured PDMS substrate. The half-cured PDMS layer facilitated the bonding with the MPTMS-treated gold pattern, which was fully cured by baking at 70 °C for 60 min. The sample was then placed on a 150 °C hotplate to weaken the TRT adhesiveness before detaching the PET carrier substrate, leaving the designed gold pattern on the PDMS substrate (Supplementary Fig. S2f).

For electrical encapsulation and selective opening of the sensor windows, a WS tape (No. 5414, 3M) was mounted on a PET film. The WS tape was also carved by the plotter cutter, leaving only ‘mask’ patterns for the exposed area (Supplementary Fig. S2g). After the transfer printing of the WS pattern onto the gold pattern (Supplementary Fig. S2h), the PDMS cover layer was coated by spinning at 3,000 rpm and baking at 70 °C for 30 min (Supplementary Fig. S2i). The sensor encapsulation was completed by dissolving the WS mask pattern in deionised water at 70 °C (Supplementary Fig. S2j).

### Fabrication and characterisation of the glucose sensor

A simple and rapid electrode functionalisation process was also developed using a plotter cutter. The mask patterns for the selective activation of PB and GOx on the WE and Ag/AgCl on the RE were created on PET films using the same plotter cutter. The masks were sequentially mounted on the fabricated sensor to selectively expose the regions to be opened (Supplementary Fig. S2k). Sequential deposition of PB/carbon paste (C2070424P2, SunChemical) on the WE and Ag/AgCl paste (011464, ALS, Japan) on the RE was performed by squeezing the paste over screen masks and curing at 100 °C, each for 5 min. The GOx layer was deposited on the PB layer using the drop-casting method. The GOx solution was prepared by mixing 1% acetic acid (695092, Sigma-Aldrich) and 2% chitosan (C3646, Sigma-Aldrich). GOx (G7141, Sigma-Aldrich; 40 mg per 1 mL of phosphate buffered saline (PBS)) and bovine serum albumin (A2153, Sigma-Aldrich; 10 mg per 1 mL of PBS) were added to the acetic acid–chitosan solution. The GOx solution (20 μL) was dropped on the WE and dried in a vacuum chamber for 20 min for a rapid even evaporation (Supplementary Fig. S2l).

## Supplementary Information

Supplementary Information.

## Data Availability

The datasets generated and/or analysed in this study are available from the corresponding author upon reasonable request.

## References

[1] Gao W (2016). Fully integrated wearable sensor arrays for multiplexed in situ perspiration analysis. Nature.

[2] Kim J, Campbell AS, de Ávila BEF, Wang J (2019). Wearable biosensors for healthcare monitoring. Nat. Biotechnol..

[3] Li Y (2017). Skin-like biosensor system via electrochemical channels for noninvasive blood glucose monitoring. Sci. Adv..

[4] Kabiri Ameri S (2017). Graphene electronic tattoo sensors. ACS Nano.

[5] Thomas K (2019). Battery-free, skin-interfaced microfluidic/electronic systems for simultaneous electrochemical, colorimetric, and volumetric analysis of sweat. Sci. Adv..

[6] Bandodkar AJ, Jeerapan I, Wang J (2016). Wearable chemical sensors: Present challenges and future prospects. ACS Sensors.

[7] Bandodkar AJ, Jeang WJ, Ghaffari R, Rogers JA (2019). Wearable sensors for biochemical sweat analysis. Annu. Rev. Anal. Chem..

[8] Kim D-H (2011). Epidermal electronics. Science.

[9] Bartholomeusz DA, Boutté RW, Andrade JD (2005). Xurography: Rapid prototyping of microstructures using a cutting plotter. J. Microelectromech. Syst..

[10] Chang BS (2019). Rapid prototyping of reconfigurable microfluidic channels in undercooled metal particle-elastomer composites. Ind. Eng. Chem. Res..

[11] Kim, H. *et al.* Simple and fast polydimethylsiloxane (PDMS) patterning using a cutting plotter and vinyl adhesives to achieve etching results. In *Proceedings of the Annual International Conference of the IEEE Engineering in Medicine and Biology Society, EMBS* 1885–1888 (2017) 10.1109/EMBC.2017.8037215. 10.1109/EMBC.2017.8037215 29060259

[12] Kim H, Seo JM (2018). Fabrication of magnetically actuated fluidic drug delivery device using polyvinyl chloride adhesive stencils. Micromachines.

[13] Yuen PK, Goral VN (2010). Low-cost rapid prototyping of flexible microfluidic devices using a desktop digital craft cutter. Lab Chip.

[14] Kojic SP, Stojanovic GM, Radonic V (2019). Novel cost-effective microfluidic chip based on hybrid fabrication and its comprehensive characterization. Sensors.

[15] Nagai M, Tada K, Shibata T (2018). Rapid prototyping of PDMS microchannels for animal and plant cells using cutting plotter and double casting. Mech. Eng. Lett..

[16] Afonso AS, Uliana CV, Martucci DH, Faria RC (2016). Simple and rapid fabrication of disposable carbon-based electrochemical cells using an electronic craft cutter for sensor and biosensor applications. Talanta.

[17] Kim HJ, Sim K, Thukral A, Yu C (2017). Rubbery electronics and sensors from intrinsically stretchable elastomeric composites of semiconductors and conductors. Sci. Adv..

[18] Gong S (2016). Fabrication of highly transparent and flexible nanomesh electrode via self-assembly of ultrathin gold nanowires. Adv. Electron. Mater..

[19] de Oliveira TR, Fonseca WT, de Oliveira Setti G, Faria RC (2019). Fast and flexible strategy to produce electrochemical paper-based analytical devices using a craft cutter printer to create wax barrier and screen-printed electrodes. Talanta.

[20] Orzari LO, de Araujo Andreotti IA, Bergamini MF, Marcolino LH, Janegitz BC (2018). Disposable electrode obtained by pencil drawing on corrugated fiberboard substrate. Sens. Actuators B Chem..

[21] Koo Y, Shanov VN, Yun Y (2016). Carbon nanotube paper-based electroanalytical devices. Micromachines.

[22] Sanger K (2017). Large-scale, lithography-free production of transparent nanostructured surface for dual-functional electrochemical and SERS sensing. ACS Sensors.

[23] de AraujoAndreotti IA (2019). Disposable and flexible electrochemical sensor made by recyclable material and low cost conductive ink. J. Electroanal. Chem..

[24] Yao S, Vargas L, Hu X, Zhu Y (2018). A novel finger kinematic tracking method based on skin-like wearable strain sensors. IEEE Sens. J..

[25] Jang NS (2017). Simple approach to high-performance stretchable heaters based on Kirigami patterning of conductive paper for wearable thermotherapy applications. ACS Appl. Mater. Interfaces..

[26] Yang S (2015). “Cut-and-paste” manufacture of multiparametric epidermal sensor systems. Adv. Mater..

[27] Yang XX (2019). “Cut-and-paste” method for the rapid prototyping of soft electronics. Sci. China Technol. Sci..

[28] Ha T (2019). A chest-laminated ultrathin and stretchable E-tattoo for the measurement of electrocardiogram, seismocardiogram, and cardiac time intervals. Adv. Sci..

[29] Xu, R. *et al.* Low-cost, efficient, photolithography-free fabrication of stretchable electronics systems on a vinyl cutter. In *Proceedings of the IEEE International Conference on Micro Electro Mechanical Systems (MEMS)* 2019-Janua, 343–346 (2019).

[30] Wang Y (2018). Low-cost, μm-thick, tape-free electronic tattoo sensors with minimized motion and sweat artifacts. npj Flexible Electron..

[31] Ameri SK (2018). Imperceptible electrooculography graphene sensor system for human–robot interface. npj 2D Mater. Appl..

[32] Dang W (2018). Stretchable wireless system for sweat pH monitoring. Biosens. Bioelectron..

[33] Eyvazi Hesar M, Khan D, Seyedsadrkhani NS, Ingebrandt S (2021). Contactless, battery-free, and stretchable wearable for continuous recording of seismocardiograms. ACS Appl. Electron. Mater..

[34] Zhou Y (2018). Multichannel noninvasive human-machine interface via stretchable μm thick sEMG patches for robot manipulation. J. Micromech. Microeng..

[35] Kao, H. L., Holz, C., Roseway, A., Calvo, A. & Schmandt, C. Duoskin: Rapidly prototyping on-skin user interfaces using skin-friendly materials. In *International Symposium on Wearable Computers, Digest of Papers* 12–16-Sept, 16–23 (2016).

[36] Shim S, Park HY, Choi GJ, Shin HC, Kim SJ (2019). A simply fabricated neural probe by laser machining of a thermally laminated gold thin film on transparent cyclic olefin polymer. ACS Omega.

[37] Imani S (2016). A wearable chemical-electrophysiological hybrid biosensing system for real-time health and fitness monitoring. Nat. Commun..

[38] Kim DH, Lu N, Ma R, Kim YS, Kim RH, Wang S, Wu J, Won SM, Tao H, Islam A, Yu KJ (2011). Epidermal electronics. Science.

[39] Lee KJ, Fosser KA, Nuzzo RG (2005). Fabrication of stable metallic patterns embedded in poly (dimethylsiloxane) and model applications in non-planar electronic and lab-on-a-chip device patterning. Adv. Funct. Mater..

[40] Byun I, Coleman AW, Kim B (2013). Transfer of thin Au films to polydimethylsiloxane (PDMS) with reliable bonding using (3-mercaptopropyl)trimethoxysilane (MPTMS) as a molecular adhesive. J. Micromech. Microeng..

[41] Zhao J (2019). A fully integrated and self-powered smartwatch for continuous sweat glucose monitoring. ACS Sensors.

[42] Bruen D, Delaney C, Florea L, Diamond D (2017). Glucose sensing for diabetes monitoring: Recent developments. Sensors.

[43] Lee H (2016). A graphene-based electrochemical device with thermoresponsive microneedles for diabetes monitoring and therapy. Nat. Nanotechnol..

[44] Bariya M, Nyein HYY, Javey A (2018). Wearable sweat sensors. Nature Electron..

[45] Kim J, Campbell AS, Wang J (2018). Wearable non-invasive epidermal glucose sensors: A review. Talanta.

[46] Adrega T, Lacour SP (2010). Stretchable gold conductors embedded in PDMS and patterned by photolithography: Fabrication and electromechanical characterization. J. Micromech. Microeng..

[47] Lacour SP, Chan D, Wagner S, Li T, Suo Z (2006). Mechanisms of reversible stretchability of thin metal films on elastomeric substrates. Appl. Phys. Lett..

