# Supplementary material for: Simple and cost-effective microfabrication of flexible and stretchable electronics for wearable multi-functional electrophysiological monitoring
Source: Sci Rep. 2021 Jul 20;11:14823. doi: 10.1038/s41598-021-94397-w (PMC8292344; doi:10.1038/s41598-021-94397-w)
Supplement: Supplementary file 1 — Supplementary Information. [file 41598_2021_94397_MOESM1_ESM.pdf]

Supplementary Information

Simple and cost-effective microfabrication of flexible and stretchable electronics for wearable multi-functional electrophysiological monitoring

Chae Hyun Kim[1], Dong Hyeon Lee[2], Jiman Youn[3], Hongje Lee[4] and Joonsoo Jeong[5,*]

[1]Medical Research Institute, Pusan National University, Yangsan, 50612, Republic of Korea

[2]School of Mechanical Engineering, Pusan National University, Busan, 46241, Republic of Korea

[3]Information Convergence Engineering, Pusan National University, Yangsan, 50612, Republic of Korea

[4]Department of Nuclear Medicine, Dongnam Institution of Radiological and Medical Sciences, Busan, 46033, Republic of Korea

[5]School of Biomedical Convergence Engineering, Pusan National University, Yangsan, 50612, Republic of Korea

[*]Corresponding author: joonsoo_jeong@pusan.ac.kr

1. Transfer printing on to medical dressing and PET film

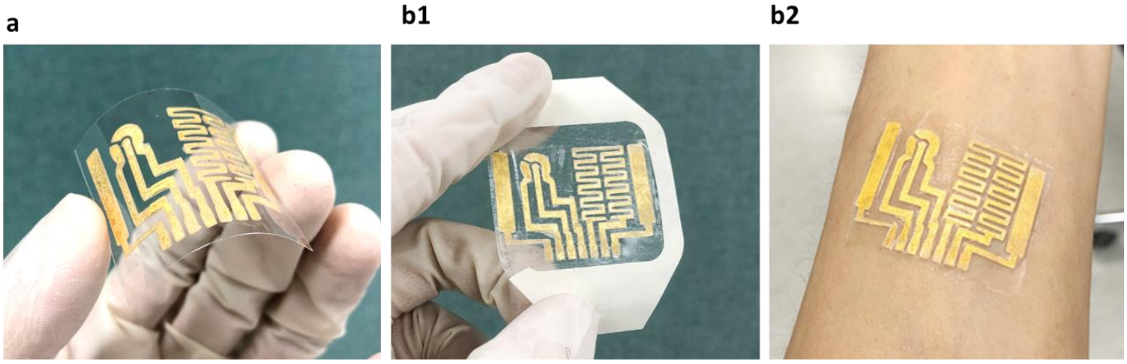

Figure S1. Photographs of the multifunctional sensor transferred onto various soft films

The multifunctional sensor pattern can be other soft materials widely used in biomedical applications using the proposed simple and rapid microfabrication process. The sensor pattern could be fabricated onto (a) 100 μm-thick PET film and (b) medical dressing (Tegaderm, 3M). (b-1) shows sensor on Tegaderm conformally attached onto skin in the forearm.

2. Detailed fabrication procedures

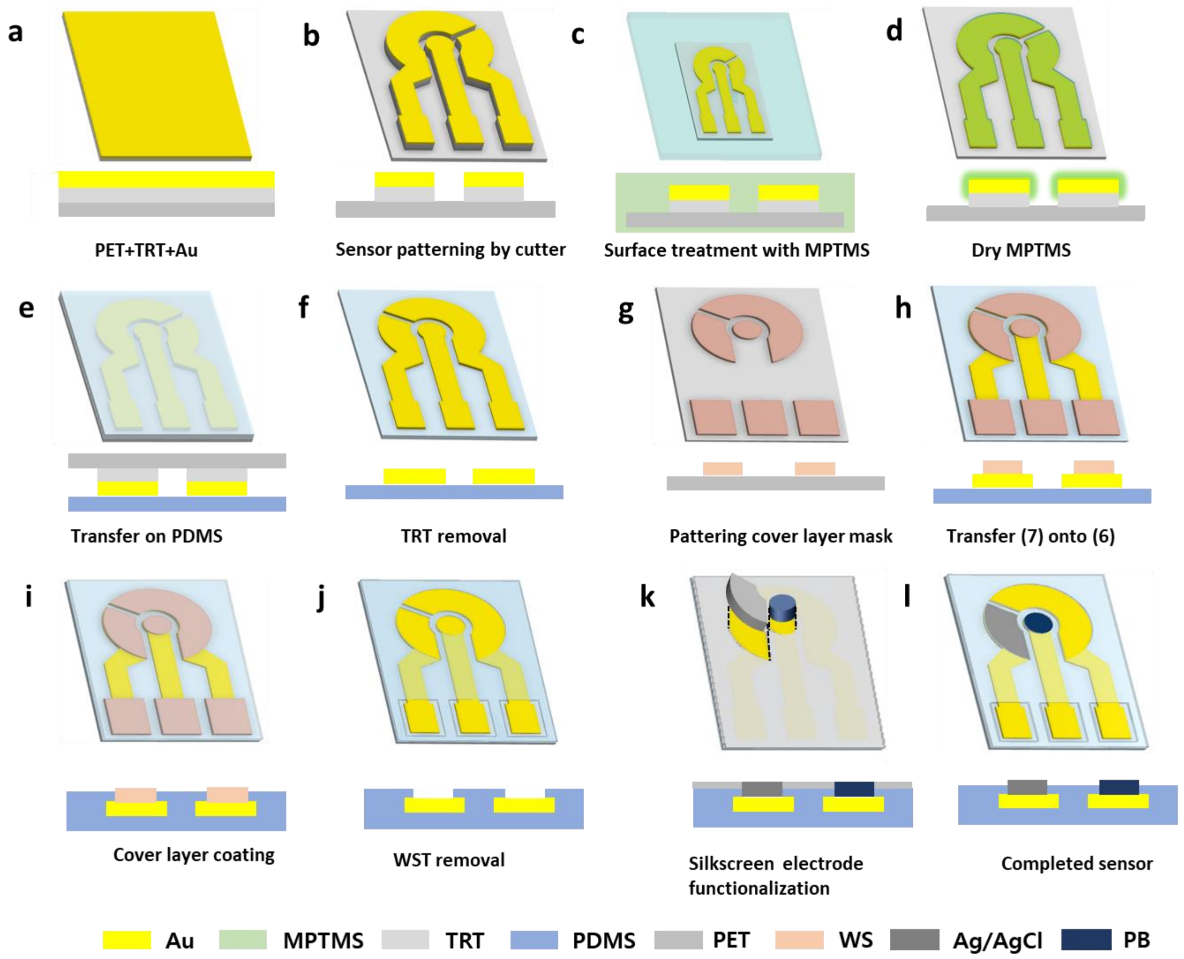

Figure S2. Schematic illustration of complete step-by-step fabrication process

Detailed description of the fabrication procedures is provided in the Methods section.

2. Calculation of “Fill factor”

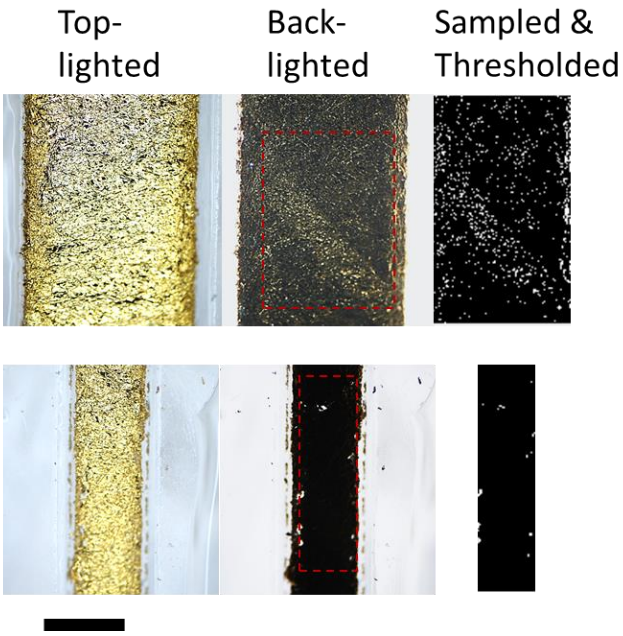

$$\text{Fill factor} = \frac{\text{Number of black pixels}}{\text{Number of black + white pixels}}$$

Figure S3. Calculation of “Fill factor” for quantification of the integrity of the patterned gold lines. (scale bar: 1 mm)

“Fill factor” was calculated as a quantified measure to assess the integrity of the fabricated gold lines by finding the number of voids which are seen as white pixels in the back-lighted ages. The original back-lighted images of the gold lines taken by stereoscopic microscope were first cropped to only contain the gold surfaces area and to trim the background area off the images. The cropped images were converted into black and white images of brightness value of 0 to 255, and threshold (>100) was applied to turn the BW images into binary images. The fill factor was then calculated by dividing (Number of all the pixels – Number of white pixels) by (Number of all the pixels).
